# Lightweight deep models based on domain adaptation and network pruning for breast cancer HER2 scoring: IHC vs. H&E histopathological images

**DOI:** 10.1371/journal.pone.0332362

**Published:** 2025-09-15

**Authors:** Lamiaa Abdel-Hamid, Pratheepan Yogarajah, Safy Hosny Ahmed Tealab

**Affiliations:** 1 Electronics and Communication Department, Faculty of Engineering, Misr International University, Cairo, Egypt; 2 School of Computing, Engineering and Intelligent Systems, University of Ulster, Londonderry, United Kingdom; 3 Pathology Department, National Cancer Institute, Cairo University, Cairo, Egypt; 4 Baheya BC Center, Cairo, Egypt; Fatih Sultan Mehmet Vakıf University: Fatih Sultan Mehmet Vakif Universitesi, TÜRKIYE

## Abstract

Human epidermal growth factor receptor 2 (HER2)-positive breast cancer is an aggressive cancer type that requires special diagnosis and treatment methods. Immunohistochemistry (IHC) staining effectively highlights relevant morphological structures within histopathological images yet can be expensive in terms of both labor and required laboratory equipment. Hematoxylin and eosin (H&E) images are more readily available and less expensive than IHC images as they are routinely performed for all patient samples. Lightweight models are well-suited for deployment on resource-constrained devices such as mobile phones and embedded systems, making them ideal for real-time diagnosis in rural regions and developing countries. In this study, IHC images are compared to H&E images for automatic HER2 scoring using lightweight deep models that incorporate several advanced techniques including network pruning, domain adaptation, and attention mechanisms. Two lightweight models are presented: PrunEff4 and ATHER2. PrunEff4 is a subset of EfficientNetV2B0 pruned to reduce the network parameters by ~80%. ATHER2 is a customized lightweight network that employs different sized convolutional filters along with a convolutional block attention module (CBAM). For PrunEff4 and ATHER2, transfer learning (pretraining on ImageNet) and domain-specific pretraining were employed, respectively. Different datasets were utilized in the development and final testing phases in order to effectively evaluate their generalization capability. In all experiments, both networks resulted in accuracies ranging from 97% to 100% for binary classifications and from 95.5% to 98.5% for multiclass classifications regardless of whether IHC or H&E images were utilized. Network pruning significantly reduced the network parameters whilst maintaining reliable performance. Domain-specific pretraining significantly enhanced performance, particularly in complex classification tasks such as HER2 scoring using H&E images and multiclass classifications. Both IHC and H&E stained images were suitable for deep learning-based HER2 scoring, given that the deep networks are efficiently trained for the specified task.

## Introduction

Breast cancer (BC) is currently the most commonly diagnosed cancer in woman worldwide [1]. In 2020, over 2.3 million new BC cases and 685,000 BC-related deaths were reported globally. By the year 2040, these numbers are predicted to rise to more than 3 million new cases and 1 million deaths per year [2]. Human epidermal growth factor receptor 2 (HER2) is a protein found on the surface of many cells, including breast cells, that controls how cells grow, repair, and divide. HER2-positive is the most aggressive BC subtype, accounting for 15% to 20% of early-stage BCs [3]. It is characterized by the amplification of the HER2 protein, leading to the excessive and rapid growth of cancer cells. HER2-positive BCs are therefore more likely to spread to other parts of the body and have worse prognosis than other BC subtypes. HER2-positive BC requires targeted medications for its effective treatment, such as trastuzumab and pertuzumab. These medications work by blocking HER2 receptors, hence slowing down or completely stopping the rapid growth of the cancer cells [4,5]. Every invasive BC should be routinely tested for the amplification of the HER2 protein in order to decide whether HER2-targeted treatment is required [6].

**Fig 1:**
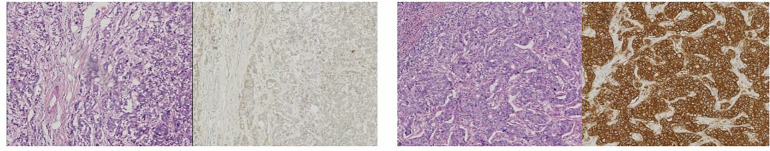
Equivalent H&E and IHC patches from the BCI dataset [13].

Histopathological analysis is the microscopic examination of tissue samples obtained from patients through biopsy to diagnose and characterize cancer. It is essential in identifying malignancies, determining tumor grade, and guiding treatment decisions. Staining techniques are used to enhance the visibility of relevant tissue structures within histopathological images, enabling more reliable diagnosis. Hematoxylin and eosin (H&E) is the gold standard staining method used in routine BC examinations [7]. Hematoxylin stains the cell nuclei in a purplish-blue color, whereas eosin stains the cytoplasm (e.g., elastic fibers, muscle fibers, and collagen) in pink shades. However, there are no morphological markers within the H&E images that can reliably predict the HER2 status. Immunohistochemistry (IHC) is a special staining method used to evaluate the HER2 status of BC [8]. IHC staining relies on two components: hematoxylin resulting in the blue coloration of the negative cells and diaminobenzidine (DAB) that gives the brown color encircling the HER2 positive tumor cells. Fig 1 shows equivalent H&E and IHC stained images.

**Fig 2:**
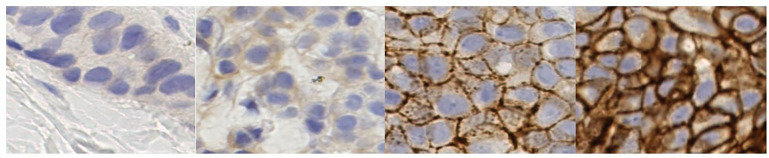
IHC BC patches of HER2 0/1+/2+/3+ cases from the Warwick dataset [14].

The American Society for Clinical Oncology and the College of American Pathologists (ASCO/CAP) guidelines state that HER2 scoring from IHC images is based on the staining intensity, the completeness of the cell membrane, and the approximate percentage of positive cells [9]. Following these guidelines, BC tumor samples can be categorized into HER2 score 0, 1+ , 2+ , or 3+ (Fig 2). Based on the ASCO/CAP 2018 guidelines, HER2 scores 0 and 1+ are considered HER2-negative, whereas a HER2 score of 3+ indicates HER2-positive BC that requires HER2-targeted treatment. HER2 score 2+ is considered a borderline case requiring further tests, e.g., Fluorescence in Situ Hybridization (FISH) and Silver in Situ Hybridization (SISH), to determine whether the cancer is HER2-positive or HER2-negative. Recent advancements of HER2 Antibody–Drug Conjugates (ADC), differentiating between HER2 score 1+ and score 0 is now considered clinically significant and can lead to different treatment plans [10]. However, current routine HER2 pathologist testing methods were primarily designed to identify HER2 overexpression, lacking specific validation methods for detecting HER2 low expression.

The manual microscopic evaluation of IHC stained slides for HER2 scoring thus requires significant medical expertise, in addition to considerable time for both preparation and examination. Furthermore, it is prone to both inter- and intra-observer variability, as its interpretation is highly subjective and depends on several factors such as the pathologist’s expertise and fatigue level as well as variations in laboratory instruments and reagents. All these factors make manual IHC scoring both time and resource intensive [11]. Computer-aided diagnosis has the potential to overcome these challenges by providing reliable and fast HER2 scoring tools using the IHC images. Several methods in literature have been developed for automatic HER2 scoring using deep learning-based approaches and were shown to give reliable performance.

H&E staining is the routine procedure for preliminary cancer detection, making them more widely available and cost efficient than IHC stained images. Interestingly, several recent studies have shown that H&E images also gave reliable performance for automatic HER2 scoring using deep networks [12]. However, most approaches relied on complex and computationally expensive models for the task [7]. Recently, edge devices have been gaining significant attention due to the rise of internet-of-medical-things (IoMT) applications, such as remote patient monitoring, wearable health trackers, smart diagnostic tools, real-time health data analysis, and telemedicine systems. Lightweight models are better suited for edge devices such as mobile phones and embedded systems. In the present study, two lightweight models are presented for automatic HER2 scoring from either IHC or H&E images. Several advanced deep learning techniques were implemented to enhance the reliability of the developed models, including network pruning, domain adaptation, and attention mechanisms. To evaluate the generalization capability of the proposed networks, two histopathological datasets from different sources were employed for their development and testing, respectively. For the two presented lightweight models, both binary and multiclass classification results are presented and compared to several state-of-the-art models from literature.

## Literature review

Deep learning methods are being increasingly used for HER2 classification. Deep networks can automatically learn both prominent and subtle relevant features from the data in an end-to-end manner, eliminating the need for manual feature engineering. Image-based deep learning methods generally rely on either standard or customized convolutional neural networks (CNNs). Standard CNNs are models that have been previously developed and vigorously tested by the machine learning community. Standard networks are commonly pretrained on a large dataset of generic images, e.g., ImageNet, before being finetuned for a specific task. This practice is commonly referred to as transfer learning (TL). On the other hand, customized CNNs are created from scratch to suit the unique characteristics of the data or the task at hand. Customized networks have the advantage of being computationally inexpensive. However, they typically require large amounts of data for their training due to being freshly initialized rather than extensively pre-trained like standard networks [15]. Table 1 summarizes relevant studies in the field, which are discussed in more detail in the next subsections. Similar to the section structure, the studies in the table are categorized according to the type of utilized histopathological images.

**Table 1:** Summary of state-of-the-art HER2 classification methods using histopathological images.

Paper	Year	Dataset	IHC/ H&E	Classes	Method	Notes	Accuracy
Tewary and Mukhopadhyay [16]	2021	Warwick	IHC	3	VGG19	TL	93.0%
Tewary and Mukhopadhyay [20]	2022	Warwick	IHC	3	AutoIHCNet	Customized Lightweight	96.0%
Wang et al. [17]	2022	TMAD	IHC	4	HER2-ResNet	Customized Lightweight	93.0%
Zheng et al. [21]	2023	Stanford	IHC	4	LMBNet	Customized Lightweight	96.9%
Mirimoghaddam et al. [18]	2024	Warwick	IHC	4	InceptionResNetV2	TL	90.5%
Kabir et al. [19]	2024	Warwick	IHC	4	ViT	TL	91.2%
Shovon et al. [24]	2022	BCI	H&E	4	HER2Net	Modified Xception	87.0%
Wang et al. [7]	2023	BCI	H&E	4	HAHNet	Customized Lightweight	93.7%
Pateel et al. [22]	2025	BCI	H&E	4	DenseNet 201, GoogLeNet, and ResNet-5	Ensemble Learning	97.4%
Mridha et al. [23]	2022	BCI	IHC H&E	4	covoHER2	Modified InceptionV3	88.0% 85.0%
L. Abdel-Hamid [24]	2025	BCI	IHC H&E	4	ConvNext	TL	97.8% 95.6%

### HER2 scoring using IHC images

IHC images are currently the gold standard for manual HER2 scoring. They have thus been primarily used for the development of deep learning-based HER2 scoring methods. Tewary and Mukhopadhyay [16] compared five standard networks pretrained on ImageNet for HER2 classification into negative (0/1+), borderline (2+), and positive (3+) which are VGG16, VGG19, ResNet50, MobileNetV2, and NASNetMobile. The VGG19 network gave the highest accuracy of 93% that increased to 98% when an image-based statistical voting mechanism was considered for whole slide image (WSI) classification. Wang et al. [17] proposed a customized HER2-ResNet network that includes two different residual units along with different size convolutional filters, batch normalization, and pooling. They compared the performance of their network to AlexNet and VGG16 for 4-class classification of HER2 using IHC images. Her2-ResNet resulted in an accuracy of 93% as compared to 89% and 75% for AlexNet and VGG16, respectively. Mirimoghaddam et al. [18] compared MobileNetV2, InceptionV3, InceptionResNetV2, to a vision transformer (ViT) and a Swin transformer. They performed experiments using real and synthetic data. They found that combining real and synthetic data resulted in highest performance and that standard CNNs significantly outperformed transformers. InceptionResNetV2 achieved the highest accuracies of 90.5% and 94.2% for the real and combined datasets, respectively. Kabir et al. [19] more recently compared GoogleNet, MobileNetV2, and DenseNet201 to a ViT for 4-class HER2 classification. Based on their presented confusion matrices, accuracies of 85.73%, 88.73%, 90.66%, and 91.15% were achieved by the four networks, respectively, indicating the usefulness of ViTs for the task.

In the scope of lightweight models, Tewary and Mukhopadhyay [20] presented the AutoIHCNet customized CNN built using three convolution blocks. They compared their method to the Xception network for 3-class HER2 classification. AutoIHCNet resulted in an accuracy of 96% whereas the Xception network achieved an accuracy of 95%. Zheng et al. [21] also presented a customized CNN which they referred to as the lightweight multi-branch network (LMBNet) for 4-class HER2 classification. They compared their results to four pretrained networks which are ResNeXt50 (~23 million parameters), EfficientNetB0 (~4 million parameters), MobileNetV3 (~4.2 million parameters), and ShuffleNetV2 (~1.26 million parameters). For the LMBNet having ~1.93 million training parameters, an accuracy of 96.92% was achieved as compared to 92.17%, 90.92%, 86.92%, and 83.92% for the considered standard networks, respectively. Both these works showed that a simple, well-designed lightweight model can outperform complex deep models for HER2 classification. However, none of these studies investigated the impact of domain-specific pretraining or the use of H&E images on HER2 scoring performance.

### HER2 scoring using H&E images

H&E stained images are more readily available than IHC images due to being part of the routine BC diagnosis pathology workflow. H&E staining does not require specialized reagents or antibodies, making it more accessible, faster to produce, and more cost efficient compared to IHC images. However, H&E-stained images are still not used for manual microscopic HER2 scoring, as disease-related structures are not visually discernible. Deep learning methods have the advantage of learning hidden structures in images in an end-to-end manner, making them an excellent candidate for HER2 scoring using H&E images. Recently, several studies have investigated the usefulness of H&E images for HER2 scoring using TL based approaches. Interestingly, these studies have shown very promising performance, as accuracies ranging from 85% to 95% were achieved in the different studies.

Wang et al. [7] introduced HAHNet which integrates Inception blocks, convolutional layers, alongside efficient channel attention modules. They compared their proposed network to seven standard networks for 4-class HER2 scoring. HAHNet gave an accuracy of 93.65% whereas the accuracies of the other networks ranged between 66% and 84%. Shovon et al. [4] presented the HE-HER2Net, which is based on the Xception network with a modified top layer. For 4-class classification, their model achieved an accuracy of 87%. HE-HER2Net outperformed several other pretrained networks such as VGG19, InceptionV3, EfficientNetB0, and DenseNet201. Pateel et al. [22] proposed an automated weighed average ensemble method referred to as HER2-ETNET that integrates the pretrained DenseNet 201, GoogLeNet, and ResNet-5 networks. Whereas 4-class classification results for each network were around 94%, the ensemble-based HER2-ETNET achieved an accuracy of 97.44%. Despite the merit of these studies, neither of them compared the results obtained from using H&E images to those IHC-stained images.

Mridha et al. [23] compared H&E and IHC images for 4-class HER2 classification. They presented the covoHER2 network, which is based on an InceptionV3 backbone with several added batch normalization and dense layers. IHC images resulted in an accuracy of 88% as opposed to an accuracy of 85% for the H&E images. In another recent study that compared H&E and IHC images for HER2 scoring [24], both image types were shown to result in comparable accuracies ranging from 95% to 98% for 4-class HER2 classification using either ConvNeXt or ResNet50. The finding that H&E images and IHC images give comparable results demonstrates the potential of using H&E images for automatic HER2 scoring. However, the indicated studies mainly relied on computationally expensive networks, making them unsuitable for resource-constrained environments.

### Domain-specific pretraining

Recently, an interesting approach emerged in literature in which a pathological dataset is used to pretrain the CNNs, as opposed to pretraining using a generic dataset such as ImageNet. The intuition is that pretraining deep networks with domain-specific images that carry similar visual features to the target dataset allows the model to learn more valuable, task-related features. This would in turn result in faster training and more reliable performance. This approach is sometimes referred to as *domain-specific pretraining*.

Ray et al. [25] compared pretraining ResNet18 and DenseNet121 networks with ImageNet as opposed to using an H&E stained colorectal cancer histopathological dataset. They evaluated their method for two BC related target classification tasks: (1) HER2 scoring using IHC images and (2) BC detection using H&E images. Experiments demonstrated that for both tasks, domain-specific pretraining resulted in slightly better performance compared to pretraining the networks with ImageNet. However, they did not evaluate their approach for the task of HER2 scoring using H&E stained images.

In the present study, two lightweight models are presented that incorporate advanced deep learning techniques such as network pruning, domain adaptation, and attention mechanisms, for automatic HER2 scoring from either IHC or H&E stained images. Specifically, our research questions are as follows:

1) Could a lightweight customized CNN pretrained on domain-specific images achieve competitive performance compared to standard deep networks pretrained on the large-scale, generic ImageNet dataset?
2) Could a lightweight standard network be further pruned to reduce the network’s complexity while maintaining reliable performance?
3) For customized and standard lightweight models, would HER2 detection using H&E images perform better than, or at least as well as, when using IHC images?

In order to investigate these research questions, two different lightweight models are introduced for automatic HER2 scoring. The first is based on pruning the lightweight EfficientNetV2B0 [26], whereas the second is a customized lightweight CNN with attention mechanisms. For more efficient training of the customized network, domain-specific pretraining is utilized where we investigate using histopathological images stained in the same and different methods for the network pretraining. In order to test the generalization capability of the proposed models, two different histopathological datasets were used for development and testing of the two presented lightweight networks, respectively. Additionally, we investigate the explainability of the two models and validate the results by consulting an expert BC pathologist with over 10 years of experience to ensure the trustworthiness of the models. Both binary and multiclass classification experiments were performed. All results were compared to state-of-the-art methods and shown to give competitive performance.

## Methods

In the present study, two lightweight deep networks are presented for automatic HER2 detection from histopathological images: ATHER2 and PrunEff4. In this section, the architecture details of these models are introduced. Next, the proposed block pruning and domain-specific pretraining methods are presented in detail. In addition, the explainable AI method employed to validate the decision making process of the presented networks is described.

### ATHER2

Histopathological images typically consist of simple geometric structures represented in two main color shades. ATHER2 (Attention to HER2) was designed as a lightweight network whose main task is to capture these structures within the histopathological images. ATHER2 has a total of 345,531 parameters, which is significantly compact compared to standard CNNs that typically have millions of parameters.

In the network’s stem, 3x3 convolution and 2x2 max pooling layers were applied to remove redundant information and downsample the input image while retaining its fine-grained details, as previously performed in Ref. [27]. ATHER2 then branches out into two distinct paths that were used to capture different relevant features from the histopathological images. In both branches, 3x3 convolutional layers and 3x3 max pooling were used to extract local patterns, edges, and texture from the downsampled feature maps. This implementation was inspired by the simple yet effective VGG network, with the modification of an increasing stride along the different stacks. This modification led to the reduction in the spatial resolution of the feature maps, hence limiting the overall network complexity.

**Fig 3:**
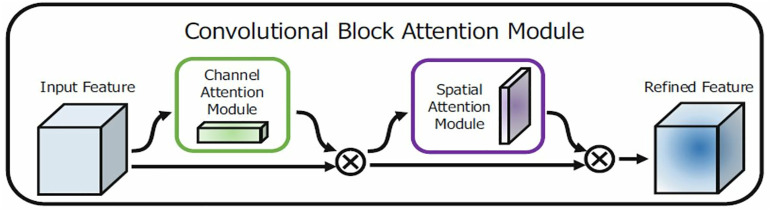
CBAM block including channel attention that identifies important channels and spatial attention that identifies important regions within the feature map [28].

ATHER2’s left and right branches differ in that the former includes the Convolutional Block Attention Module (CBAM), whereas the latter employs a 5x5 convolutional layer. In the left branch, the CBAM block [28] was used to improve performance by incorporating both channel and spatial attention modules which help the network focus on important regions in the feature maps (Fig 3). Specifically, channel attention identifies the important channels, whereas spatial attention identifies the important regions within the feature map. In addition, a dropout layer was added for regularization purposes. As for the right branch, a 5x5 convolutional layer was used to capture information from a broader area in the feature maps, which is especially useful in recognizing the larger patterns in the histopathological images. In addition, batch normalization was added for more efficient network training. At the end of each branch, Global average pooling (GAP) was applied to reduce the number of parameters while encouraging the network to focus on the most informative and discriminative regions. GAP also serves as a form of spatial regularization that helps prevent overfitting. Finally, dense layers were added to combine the features extracted from the different convolutional layers for the final predictions. A sigmoid or softmax activation layer was used in the final dense layer for binary or multiclass classification, respectively.

**Fig 4:**
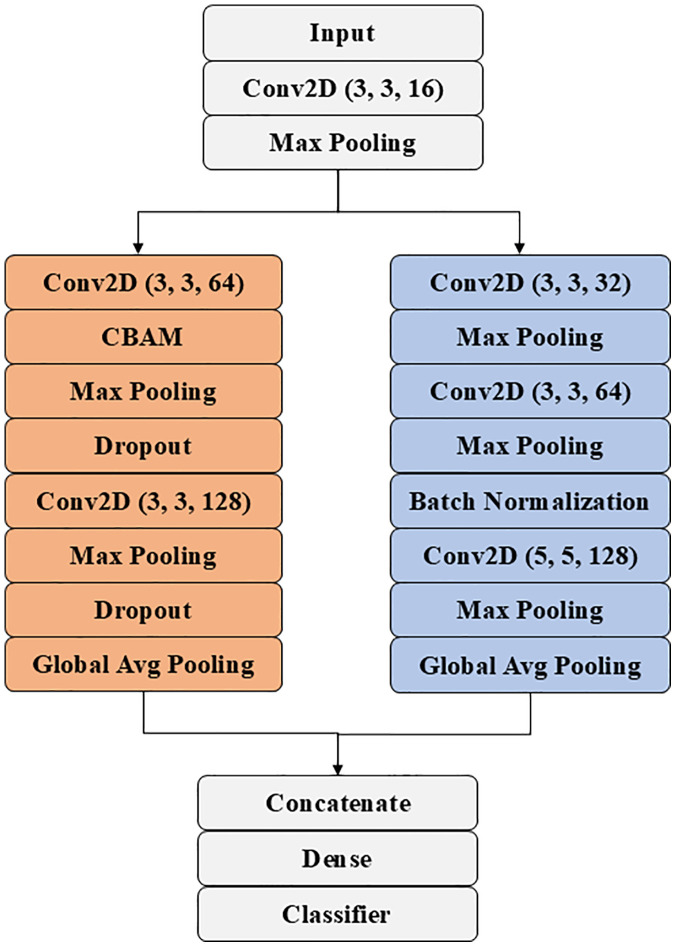
ATHER2 block diagram comprising two branches: the left branch utilizes 3x3 convolutional filters combined with CBAM attention and the right branch considers different sized convolutional filters along with batch normalization. In both branches, max pooling is employed to reduce the spatial dimensions of the feature map for reduced computational complexity.

Leaky rectifier linear unit (ReLU) activation functions were utilized throughout the ATHER2 network. Leaky ReLU is similar to the ReLU function except that it returns small negative outputs proportional to the neuron input instead of directly giving a zero output for the negative values. Leaky ReLU thus prevents the total discard of potentially useful information. This ensures that all neurons in the network contribute to the output which leads to a more generalized performance. In order to further increase the network’s generalization capability, L2 regularization was implemented which works by adding the sum of the squared values of the model coefficients to the cost function thus encouraging smaller but non-zero weights. L2 regularization constrains the model’s complexity, promoting smoother weight distributions and reducing sensitivity to noisy or irrelevant features in the training data. ATHER2’s architecture is shown in Fig 4 and its details are summarized in Table 2.

**Table 2:** ATHER2 network architecture.

	Layer	Description	Output Size
C-0	**Input**	Image size = 512 × 512 × 3	512 × 512 × 3
C-1	**Convolution**	size = 3 × 3 × 16, stride = 1	512 × 512 × 16
C-2	**Max Pooling**	pool size = 2 × 2, stride = 2	256 × 256 × 16
L-1	**Convolution + CBAM**	size = 3 × 3 × 64, stride = 3	86 × 86 × 64
L-2	**Max Pooling**	pool size = 3 × 3, stride = 2	42 × 42 × 64
L-3	**Dropout**	10%	42 × 42 × 64
L-4	**Convolution**	size = 3 × 3 × 128, stride = 3	14 × 14 × 128
L-5	**Max Pooling**	pool size = 3 × 3, stride = 2	6 × 6 × 128
L-6	**Dropout**	10%	6 × 6 × 128
L-7	**GAP**	--	128
R-1	**Convolution**	size = 3 × 3 × 32, stride = 1	256 × 256 × 32
R-2	**Max Pooling**	pool size = 3 × 3, stride = 1	254 × 254 × 32
R-3	**Convolution**	size = 3 × 3 × 64, stride = 2	127 × 127 × 64
R-4	**Max Pooling**	pool size = 3 × 3, stride = 2	63 × 63 × 64
R-5	**Batch normalization**	--	63 × 63 × 64
R-6	**Convolution**	size = 5 × 5 × 128, stride = 2	32 × 32 × 128
R-7	**Max Pooling**	pool size = 3 × 3, stride = 3	10 × 10 × 128
R-8	**GAP**	--	128
C-3	**Concatenate**	--	256
C-4	**Dense**	size = 128, activation = ‘Leaky ReLU’	128
C-5	**Dense**	size = 1, activation = ‘Sigmoid’	1

### EfficientNet

EfficientNet [29] is a family of CNNs that was inspired by the MobileNetV2 architecture [30]. MobileNet was among the early lightweight models specifically designed for resource-constrained environments such as mobile phones and embedded devices. EfficientNets builds on this efficiency by introducing a compound scaling method that balances network depth, width, and resolution while maintaining or improving performance. EfficientNets are hence a family of models that achieve higher accuracy with fewer parameters.

Mobile Inverted Bottleneck Convolution (MBConv) blocks, originally introduced in MobileNetV2, are considered the main building blocks of the EfficientNet family. The MBConv block is a mobile inverted residual block with additional Squeeze and Excitation (SE) mechanism. Residual blocks introduce skip connections that link the input and output of a convolutional block, enabling the network to preserve and reuse unmodified feature representations from earlier layers. Residual blocks thus improve training and eliminate the problem of vanishing gradients in extremely deep networks. Typically, residual blocks follow a wide → narrow → wide approach. Inverted residuals, on the other hand, follow a narrow → wide → narrow approach such that the number of channels increases at the bottleneck. Inverted residual blocks are considered more computationally efficient than traditional residual blocks as they have significantly fewer parameters. Finally, an SE block [31] is integrated within the MBConv block in order to enhance the representational power and efficiency of the network.

**Fig 5:**
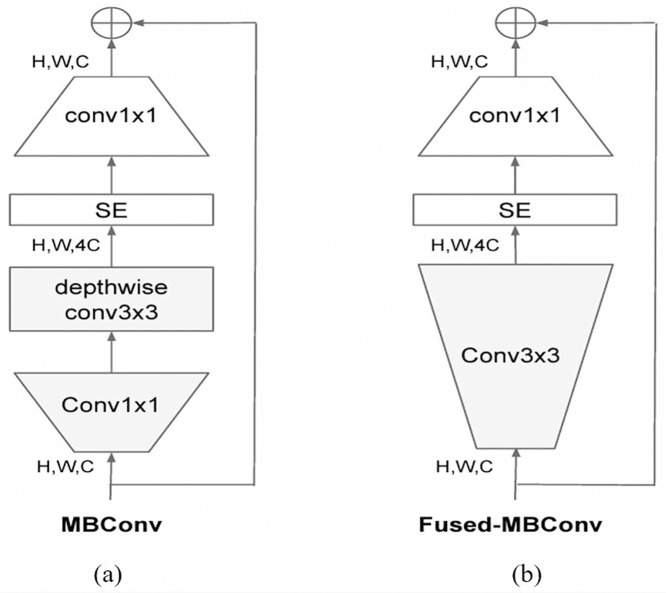
Block diagrams of (a) MBConv originally introduced in MobileNetV2 and adopted in EfficientNet, (b) Fused-MBConv later implemented in EfficientNetV2 to improve training efficiency [22].

EfficientNetV2 [26] is an updated and more compact version of the EfficientNet family introduced later by the same authors. EfficientNetV2 builds upon the main blocks presented in the original EfficientNet but introduces improvements in scaling strategies and architectural design to achieve better efficiency and performance. Among the major distinctions between them is that EfficientNetV2 extensively uses the fused-MBConv alongside the original MBConv in the early layers. The fused-MBConv replaces the depthwise conv3 × 3 and expansion conv1 × 1 in MBConv with a single regular conv3 × 3 (Fig 5). EfficientNetV2 also completely removes the last stride-1 stage in the original EfficientNet due to its memory access overhead and large parameter size. These tweaks allowed EfficientNetV2 to significantly outperform its predecessors on the benchmark ImageNet dataset. The EfficientNetV2 family consists of eight variations from B0 to B7, where larger indices indicate variants with larger number of parameters and resulting in higher ImageNet accuracy.

**Fig 6:**
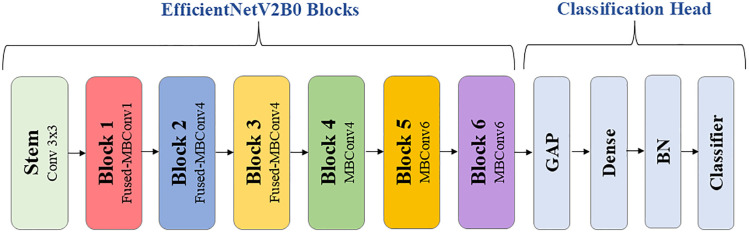
EfficientNetV2B0 simplified architecture showing its six different blocks and the newly added classification head.

In the present study, EfficientNetV2B0 was considered for HER2 classification from histopathological images. EfficientNetV2B0 is the most compact model offering a good balance between performance and computational requirements (Fig 6). EfficientNetV2B0 has approximately 7.2 million parameters, whereas InceptionV3, ResNet50, and VGG16 have about 23.8, 25.6, and 138.4 million parameters, respectively. EfficientNetV2B0 thus has considerably fewer parameters than other state-of-the-art standard deep networks making it far more suitable for devices with limited computational resources [31].

### Block pruning

Generally, model pruning is a method used to remove redundant network parameters for the benefit of reducing the network size without significantly compromising performance. Model pruning is widely used to reduce the hardware requirements of deep models in low resource settings [32]. In this work, a novel model pruning method is introduced where the top network blocks are removed to reduce the size and complexity of a standard convolutional network. This technique will be referred to as ***block pruning***. Block pruning is introduced under the intuition that in very deep standard networks, top blocks are intended to capture high level features, such as faces and objects. High level features are not present in histopathological images, which basically consist of simple, low level geometric structures. Consequently, removing the upper network layers is performed to reduce computational complexity without affecting the network’s low-feature extraction capabilities, thus maintaining overall reliable performance.

In this work, block pruning was applied to EfficientNetV2B0 where its top blocks were subsequently removed, and each modified subnetwork’s performance was evaluated. Since EfficientNetV2B0 reportedly has six different blocks, block pruning resulted in five different subnetworks of diminishing sizes. In order to enhance feature extraction, a convolution filter having a kernel size of 7 and 192 channels is added before the classification head in all the pruned versions. This allow the model to project features into a richer representation space before the final classification.

**Fig 7:**
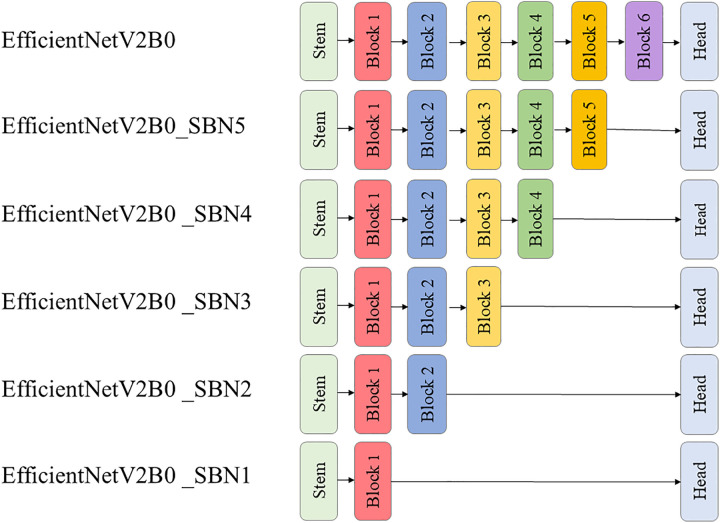
EfficientNetV2B0 and its five pruned subnetworks.

Fig 7 shows the simplified architecture of the original EfficientNetV2B0 in comparison to its five pruned subnetworks. Table 3 summarizes the number of parameters for the original EfficientNetV2B0 and its five pruned subnetworks. In the table, the stop layer refers to the name of the final layer after which the network was pruned to create the subnetwork. The original EfficientNetV2B0 has ~ 7.2 million parameters. Each block removed subsequently almost halved the number of parameters. The customized ATHER2 network has a total of 345,531 parameters making it comparable in complexity to the smallest EfficientNetV2B0 subnetwork that has approximately 358k parameters.

**Table 3:** Number of parameters in EfficientNetV2B0 as compared to its pruned subnetworks and ATHER2.

Name	Blocks	Stop Layer	No. of layers	Trainable parameters	Non-trainable parameters	Total parameters
**EfficientNetV2B0**	**ALL**	*none*	274	7,177,108	63,168	**7,240,276**
**EfficientNetV2B0_SBN5**	**1-5**	*block5e_add*	154	2,628,008	20,416	**2,648,424**
**EfficientNetV2B0_SBN4**	**1-4**	*block4c_add*	81	1,530,432	6,240	**1,536,672**
**EfficientNetV2B0_SBN3**	**1-3**	*block3b_add*	38	848,196	1,824	**850,020**
**EfficientNetV2B0_SBN2**	**1-2**	*block2b_add*	26	561,668	992	**562,660**
**EfficientNetV2B0_SBN1**	**1**	*block1a_project_activation*	14	358,404	480	**358,884**
**ATHER2**	**--**	**--**	24	345,401	130	**345,531**

### Transfer learning vs. domain-specific pretraining

Deep networks require huge amounts of data for their robust training. This can be a challenge in the medical domain. Large annotated medical datasets are considerably scarce due to several factors such as patient privacy issues and the extensive workload associated with the dataset labelling. In order to overcome these limitations, TL has been widely adopted in medical classification problems to facilitate the robust training and improved generalization of CNNs without the need for excessively large datasets. TL involves pretraining a standard deep network on a large generic dataset (upstream task), then finetuning the pretrained network using the small target dataset (downstream task). ImageNet [33] is the benchmark dataset typically employed in the pretraining of deep standard networks. ImageNet consists of approximately 1.2 million images in 1,000 different classes taken from WordNet, such as flamingo, strawberry, and bird house.

In the field of medical image classification, standard deep networks pretrained on ImageNet lead to significant performance improvements relative to the freshly initialized versions of the same networks. The intuition of this approach is that the first layers within convolutional networks extract low-level features such as lines, edges, and color, which are common to all types of images, whether medical or non-medical. However, the characteristics of ImageNet images are quite different from those of medical images [34,35]. In order to overcome this issue, another approach has emerged in literature in which pretraining is accomplished using a dataset coming from a domain closely related to the required downstream task [25,36,37]. This approach is commonly referred to as *domain-specific pretraining*.

**Fig 8:**
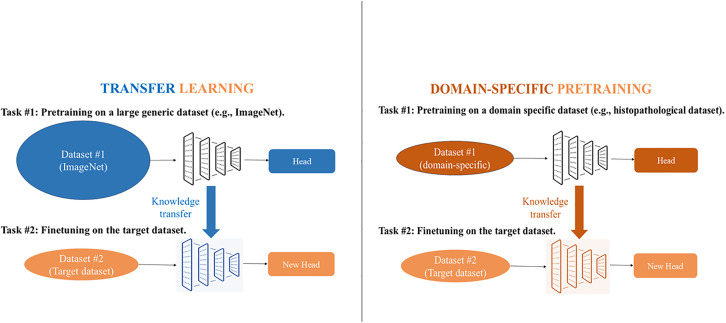
Transfer learning vs. domain-specific pretraining workflows.

In a sense, domain-specific pretraining shares the same basic concept of TL; however, instead of utilizing a generic dataset such as ImageNet, the model is pretrained on a dataset that closely resembles the target dataset. This approach ensures that the model learns features that are more related to the target task, thus leading to more efficient training and improved performance. And since the features learned during pretraining closely resemble those of the target task, the pretraining dataset needn’t be as large as in traditional TL methods. This is particularly relevant because medical classification tasks are generally less complex than general computer vision tasks, that typically include distinguishing between tens or hundreds of diverse and unrelated objects. Fig 8 summarizes the TL and domain-specific pretraining workflows.

In histopathological analysis, different staining agents are used to highlight the relevant structures in biopsy samples depending on the required diagnosis. IHC stained images used for HER2 scoring display blue and brown colors. On the other hands, H&E stained images used for BC detection display purplish-blue and pinkish colors. Nevertheless, regardless of the color scheme, both IHC and H&E histopathological images have quite similar geometric structures. Accordingly, a model pretrained on one type of stained images is expected to perform well when fine-tuned on the other type of stained images. In the present study, domain-specific pretraining is utilized to improve the training of the customized ATHER2 network.

### Grad-CAM

Computer-aided diagnosis tools are expected to be trustworthy, transparent, and interpretable in order to earn the approval of healthcare professionals [27]. Despite the substantial and ground-breaking performance achieved by deep networks in various fields, it is not always easy to comprehend the decision-making process of these models. Deep networks are considered ‘*black* *boxes’* as they suffer from lack of explainability. Recently, there has been an increasing interest in understanding why a deep network makes a specific decision. In the medical domain, this information can be revised by medical experts to validate the interpretability and trustworthiness of the models. Explainable artificial intelligence (XAI) thus has the merit of increasing the trust of medical experts in the diagnosis performed by the AI models.

Gradient-weighted class activation mapping (Grad-CAM) [38] is a popular XAI technique that helps us understand why a CNN makes a certain decision. Grad-CAM works by passing an image through a CNN until it reaches the target layer of interest. These upper layers are typically chosen as they tend to contain the most discriminative features [39]. Subsequently, the gradients for the target class are calculated with respect to the feature maps of the chosen convolutional layer using backpropagation. GAP is then applied followed by a weighed combination of the forward activation maps. Next, a ReLU function is applied to emphasize the regions that positively influenced the classification decision. Finally, Grad-CAM provides a heatmap in which the most influential regions in the model’s decision-making process are highlighted on the input image.

In this work, the Grad-CAM technique was implemented to investigate the explainability of the two presented lightweight networks. An expert pathologist with over 10 years of experience reviewed the regions highlighted by the Grad-CAM technique, to assess whether the networks were focusing on the relevant structures within the histopathological images. This approach was adopted to ensure the reliability of the two proposed lightweight models for HER2 scoring in BC diagnosis.

## Results and discussion

Two lightweight deep networks are proposed in this work for HER2 scoring using histopathological images, EfficientNetV2B0 and ATHER2. In this section, we first present the details of the BCI and Warwick datasets utilized for the development and testing of the proposed models, respectively. The BreakHis dataset used for the domain-specific pretraining of the EfficientNetV2B0 is also introduced. Next, the hyperparameter tuning experiments are illustrated and a summary of the considered parameters is provided. Finally, the results of the different experiments performed for the development and testing of the two introduced lightweight networks are presented and discussed in relation to previous literature.

All model implementations and experiments were conducted using the Keras framework in Python on a computer with an Intel Core i7-11800H CPU @2.3 GHz, 16 GB RAM, and an 8 GB GPU (GeForce RTX3060).

### Datasets

Three public histopathological datasets were considered in this work. Primarily, the BCI dataset was used for the development of the introduced lightweight models for automatic HER2 scoring. In the final experiments, the Warwick dataset was utilized to test the generalization capability of the proposed models through evaluating their performance on an unseen dataset. The BreakHis dataset was solely utilized for the domain-specific pretraining of the ATHER2 network. All datasets were directly considered without applying any preprocessing techniques to the images. The details of the three histopathological datasets are as follows:

1) **BC Immunohistochemical (BCI)** [13] includes equivalent IHC and H&E image patches taken from the WSI pairs of 51 BC patients. The BCI dataset was introduced in an image-to-image translation competition that required the generation of IHC images from their corresponding H&E images. Accordingly, some image patches barely contain any relevant cell structures but are predominantly noisy patches. Dataset cleaning was performed to remove noisy patches as was previously performed in Refs. [14] and [40]. The BCI dataset considered in this work includes 3,421 IHC/H&E image pairs having a resolution of 1024x1024 pixels. The train and test datasets originated from different patients in order to ensure reliable performance assessment [41]. Table 4 presents the number of images in each class for the training and testing data.
2) **Warwick dataset** [42] was introduced by the Tissue Image Analytics Center (TIA) at the University of Warwick, United Kingdom. The dataset includes 172 WSIs taken from 86 cases of invasive BC and includes IHC and H&E stained slides. In this work, we adopt the version of the Warwick dataset presented in Ref. [14] which includes IHC image patches captured from 52 WSI images at x40 magnification. In this version, the patches with significant tissue information were automatically identified by analyzing the patch histograms. Assisted by an expert pathologist, the most representative 30 patches were then selected for each patient. In total, the dataset consists of 1,504 IHC patches divided as follows: score 0: 278 images, score 1 + : 387 images, score 2 + : 419 images, and score 3 + : 420 images. All patches have a resolution of 250x250 pixels. In this work, two experiments were conducted using the Warwick dataset. In the first experiment, the train and test images were randomly selected. In the second experiment, a subject-wise split was performed such that the train and test dataset included images from completely different patients. The second experiment more accurately evaluates the model’s ability to generalize to unseen patients, hence will be used for the final evaluation of the proposed lightweight models.
3) **BreakHis dataset** [43] was provided by the Laboratory of Vision Robotics and Imaging at the Federal University of Parana, Brazil. BreakHis originally consists of 625 benign and 1,370 malignant H&E images taken from the WSI of 82 patients. In this work, a balanced subset of the BreakHis dataset was utilized that consists of 625 benign images along with 672 malignant images randomly selected from the original dataset. All images were captured at a magnification factor of x40 and have a resolution of 700x400 pixels. This dataset was solely used for the domain-specific pretraining of the introduced ATHER2 network in the different experiments.

**Table 4:** BCI dataset details.

		Train	Test	Total
**Negative**	0	172	23	1,101
	1+	804	102	
**Borderline**	2+	1,418	187	1,605
**Positive**	3+	620	95	715
**Total**		**3,014**	**407**	**3,421**

### Hyperparameter tuning

For both EfficientNetV2B0 and ATHER2, an Adam optimizer was used with a learning rate scheduler which decreased the initial learning rate by a factor of ten when performance hit a plateau. Learning rate scheduling allows the deep networks to start training at relatively large values, facilitating their convergence in the initial training stages. The learning rate is then decreased at later stages to help fine-tune the model’s parameters and prevent it from overshooting the optimal solution. The initial learning rate was empirically chosen to be a value of either 6x10^-2^, 6x10^-3^, or 6x10^-4^ where larger learning rates were adopted for freshly initialized networks whereas smaller values were employed when fine tuning the deep networks. A maximum of 80 epochs was considered in all experiments.

Early stopping and data augmentation were implemented as regularization techniques to enhance the performance and robustness of the trained models. Early stopping is based on the intuition that neural networks will overfit beyond a certain training point. Accordingly, the validation accuracy is monitored and training stops when it ceases to improve over several epochs. Data augmentation is a technique used to increase the diversity of the training dataset by introducing slightly modified copies of the existing data. Data augmentation results in more robust training, consequently improving the generalization capability of the trained networks. In this work, vertical and horizontal flipping along with width and height shift were applied to the training datasets using the *ImageDataGenerator* in TensorFlow [44].

Batch size and input image resolution were determined by performing HER2 positive/negative binary classification experiments in order to find the optimal values that maximize the model’s accuracy. For these experiments, the BCI dataset’s IHC images were utilized. Experimental results, summarized in Tables 5–7, showed that larger batch sizes and image resolutions resulted in better overall performance. For ATHER2, an input image resolution of 512x512 pixels and a batch size of 24 were selected. For EfficientNetV2B0, a batch size of 24 was also chosen as it was shown to give the highest overall performance, yet the default input image resolution of 224x224 pixels was considered due to hardware limitations. Table 8 summarizes the hyperparameter values considered in the present study. It is worth stating that for EfficientNetV2B0, all layers were set as trainable in order to effectively finetune the pretrained network layers on the new target task comprising the histopathological images. Since the upstream and downstream datasets come from significantly different domains, ImageNet vs. histopathological images, this approach was found to give consistently better performance than freezing all or part of EfficientNetV2B0’s layers during training.

**Table 5:** Effect of batch size on the performance of ATHER2 (BCI dataset – IHC images – 2 classes).

Batch Size	Accuracy	Precision	Recall	F1-Score	AUC
24	**98.64**	**100.0**	**96.84**	98.39	**99.83**
16	98.18	98.92	96.84	97.87	99.75
8	97.73	98.91	95.79	97.33	99.44
4	98.18	98.92	96.84	97.87	99.34

**Table 6:** Effect of batch size on the performance of EfficientNetV2B0 (BCI dataset – IHC images - 2 classes).

Batch Size	Accuracy	Precision	Recall	F1-Score	AUC
**24**	**99.07**	**98.90**	**98.90**	**98.90**	**99.94**
16	99.04	98.80	98.80	98.80	99.98
8	99.07	100.0	97.80	98.89	99.77
4	98.64	98.94	97.89	98.41	99.79

**Table 7:** Effect of input image size on performance of ATHER2 (BCI dataset – IHC images – 2 classes).

Image Resolution	Accuracy	Precision	Recall	F1-Score	AUC
**512x512**	**98.64**	**100.0**	**96.84**	**98.39**	**99.83**
384x384	98.18	98.92	96.84	97.87	99.64
224x224	98.18	98.92	96.84	97.87	99.70
128x128	96.82	97.83	94.74	96.26	98.93
96x96	96.36	95.79	95.79	95.79	97.75

**Table 8:** Summary of hyperparameters.

Hyperparameter	Value
Image Size	ATHER2: 512x512 EfficientNet: 224x224
Optimizer	Adam
Learning Rate	LR Scheduler
Batch Size	24
Loss Function	Cross Entropy

### Experimental results

Four different experiments were performed in order to develop and test the two proposed lightweight networks:

1) **HER2 positive/negative classification using IHC images (BCI Dataset):** Experiments were performed to investigate the performance of different pruned versions of EfficientNetV2B0. Additionally, the effect of domain-specific pretraining on the performance of ATHER2 was explored.
2) **HER2 positive/negative classification using H&E images (BCI Dataset):** The performance of PrunfEff4 – the pruned version of EfficientNetV2B0 - was compared to ATHER2 considering H&E images.
3) **HER2 multiclass classification using IHC and H&E images (BCI Dataset):** 3- and 4- multiclass classifications were performed using the BCI dataset. Results were compared to state-of-the-art methods from literature for the same dataset.
4) **HER2 multiclass classification using IHC images (Warwick Dataset):** Warwick dataset, which has not been previously seen by either PrunEff4 or ATHER2, was used to test the generalization capability of the networks. Additionally, H&E benign/malignant images (BreakHis) and IHC HER2-positive/Her2-negative images (BCI dataset) were compared for the domain-specific pretraining of the customized ATHER2. Final results for all experiments were compared to state-of-the-art methods from literature for the same dataset.

#### HER2 binary classification using IHC images (BCI Dataset).

HER2 binary classification is performed to distinguish between HER2-positive (3+) and HER2-negative (0/1+) classes. The main aim of this analysis is to compare the performance of different pruned versions of EfficientNetV2B0 in order to find the most efficient subnetwork for the target task. Additionally, ATHER2 network was evaluated in two scenarios, when freshly initialized and when pretrained on the BreakHis dataset. All experiments were performed using the BCI dataset’s IHC images.

Table 9 summarizes the classification results of the four classification experiments: (a) EfficientNetV2B0 network and its pruned versions freshly initialized, (b) EfficientNetV2B0 network and its pruned versions pretrained on ImageNet (TL), (c) ATHER2 freshly initialized, and (d) ATHER2 pretrained on BreakHis (domain-specific pretraining). For each of the four experiments, the best results are highlighted in bold.

**Table 9:** HER2 binary classification performance for the (a) EfficientNetV2B0 subnetworks, (b) EfficientNetV2B0 subnetworks pretrained on ImageNet, (c) ATHER2, and (d) ATHER2 pretrained on BreakHis (BCI dataset – IHC images).

Network	Pretraining	Accuracy	Precision	Recall	F1-Score	AUC
**EfficientNetV2B0**	**No**	97.69	97.78	96.70	97.24	99.69
**EfficientNetV2B0_SBN5**		98.15	100.0	95.60	97.75	99.70
**EfficientNetV2B0_SBN4 (PrunEff4)**		**98.15**	**98.88**	**96.70**	**97.78**	**99.43**
**EfficientNetV2B0_SBN3**		97.69	98.86	95.60	97.20	99.75
**EfficientNetV2B0_SBN2**		97.69	97.78	96.70	97.24	99.56
**EfficientNetV2B0_SBN1**		97.69	97.78	96.70	97.24	99.82
**EfficientNetV2B0**	**ImageNet**	99.07	98.90	98.90	98.90	99.94
**EfficientNetV2B0_SBN5**		99.07	100.0	97.80	98.89	99.93
**EfficientNetV2B0_SBN4 (PrunEff4)**		**100.0**	**100.0**	**100.0**	**100.00**	**100.0**
**EfficientNetV2B0_SBN3**		99.07	100.0	97.80	98.89	99.91
**EfficientNetV2B0_SBN2**		98.61	97.83	98.90	98.36	99.85
**EfficientNetV2B0_SBN1**		97.69	97.78	96.70	97.24	99.77
**ATHER2 – Left Branch**	**No**	94.55	95.60	95.18	95.39	97.61
**ATHER2 – Right Branch**		98.18	98.92	96.84	97.87	99.69
**ATHER2 – Full Network**		**98.64**	**100.0**	**96.84**	**98.39**	**99.83**
**ATHER2 – Full Network**	**BreakHis**	**98.64**	**98.64**	**97.89**	**98.26**	**99.55**

**Block pruning results:** For EfficientNetV2B0, all the ImageNet pretrained networks outperformed the freshly initialized ones, although in most cases the improvement was less than 3%. This observation shows that the HER2 classification task could be simple enough to be solved without the need for extensive pretraining. Notably, the comparable performance of the freshly initialized and pretrained EfficientNetV2B0 models served as a key motivation for designing a new lightweight customized network, as well as for the EfficientNetV2B0 pruning.

Results from the different pruned networks indicate that subsequently removing the top two blocks had almost no effect on performance whereas further pruning led to degraded performance. For both freshly initialized and pretrained networks, EfficientNetV2B0_SBN4 achieved the highest performance among the pruned subnetworks. Specifically, the pretrained EfficientNetV2B0_SBN4 resulted in accuracy and AUC of 100%, whereas its freshly initialized version resulted in accuracy and AUC of 98.15% and 99.43%, respectively. These results indicate that the full complexity of the EfficientNetV2B0 network can be considered unnecessary for reliable HER2 score detection from the IHC histopathological images.

**Domain adaptation results:** For the ATHER2 network, two cases were considered which are freshly initialized and domain-specific pretraining. In the latter case, ATHER2 was pretrained on the balanced BreakHis dataset which includes H&E images classified as benign or malignant BC cases. ATHER2 network resulted in an accuracy of 98.64% for both the freshly initialized and the domain-specific pretraining experiments.

An interesting observation is that the domain-specific pretraining of the ATHER2 network had a minimal effect on the HER2 binary classification performance when using the IHC images. IHC images are the gold standard for HER2 scoring as they have prominent HER2 related structures that can be easily captured by well-developed deep convolutional networks [46]. Consequently, ATHER2 was able to successfully capture the HER2 related structures within the IHC images, regardless of whether or not domain-specific pretraining was utilized. Nevertheless, more challenging scenarios are expected to better benefit from domain-specific pretraining such as multiclass classifications or when H&E images are adopted for HER2 classification. Given that H&E images lack the explicit HER2 related structures present in IHC images, the deep models must learn to infer HER2 expression from subtler morphological patterns. Pretraining ATHER2 on a domain-related dataset can thus enhance the model’s ability to capture these subtle features, leading to improved HER2 classification performance. These scenarios will be thoroughly investigated in the next subsections.

An ablation study was performed in which only one of the ATHER2 branches was removed while the other was used for classification. The left branch resulted in an accuracy of 94.55% whereas the right branch resulted in an accuracy of 98.18%. The full ATHER2 network resulted in an accuracy of 98.64%, which is comparable to performance of the pretrained networks. These results indicate that implementing the full ATHER2 network resulted in a more reliable performance than when only a single branch was considered for the HER2 classification.

This is not the first attempt in literature that showed that a well-devised lightweight customized network performs just as well as pretrained standard networks. Dwivedy et al. [47] proposed LMNet which is a lightweight deep convolutional network for COVID19 detection from X-ray images. LMNet is composed of a stack of convolutional filters of different sizes namely 7, 5, 3, and 1. LMNet had slightly less than 1 million parameters and achieved results that were comparable to several standard CNNs pretrained on ImageNet including VGG19, InceptionV3, Xception, DenseNet169, and MobileNetV2. Abdel-Hamid [48] presented a two-branched deep convolutional network (TWEEC) for glaucoma detection from retinal images. TWEEC used 9x9 convolutional filters with average pooling in one branch, and 3x3 convolutional filters with max pooling in the other branch in order to extract features relevant to the retinal image’s luminance and blood vessels, respectively. Experiments showed that TWEEC achieved an accuracy of 98.78%, hence outperforming several standard and customized deep networks by 8–15%.

In conclusion, ATHER2 gave comparable performance to those attained by the standard EfficientNetV2B0 and its pruned EfficientNetV2B0_SBN4 subnetwork with the advantage of ATHER2 having significantly less parameters (ATHER2: ~ 345K parameters, EfficientNetV2B0_SBN4: ~ 1.5M parameters, and EfficientNetV2B0: ~ 7.2M parameters). Interestingly, the ATHER2 network also achieved better performance than its close sized EfficientNetB0_SBN0 subnetwork, where ATHER2 gave an accuracy of 98.64% whereas EfficientNetV2B0_SBN0 gave an accuracy of 97.69%. For the rest of this work, only EfficientNetV2B0 and EfficientNetV2B0_SBN4 pretrained on ImageNet will be considered alongside ATHER2 as they were shown to give the most robust performance. For simplicity, the pruned network EfficientNetV2B0_SBN4 will be referred to as the *PrunEff4* model for the remainder of this paper.

**Fig 9:**
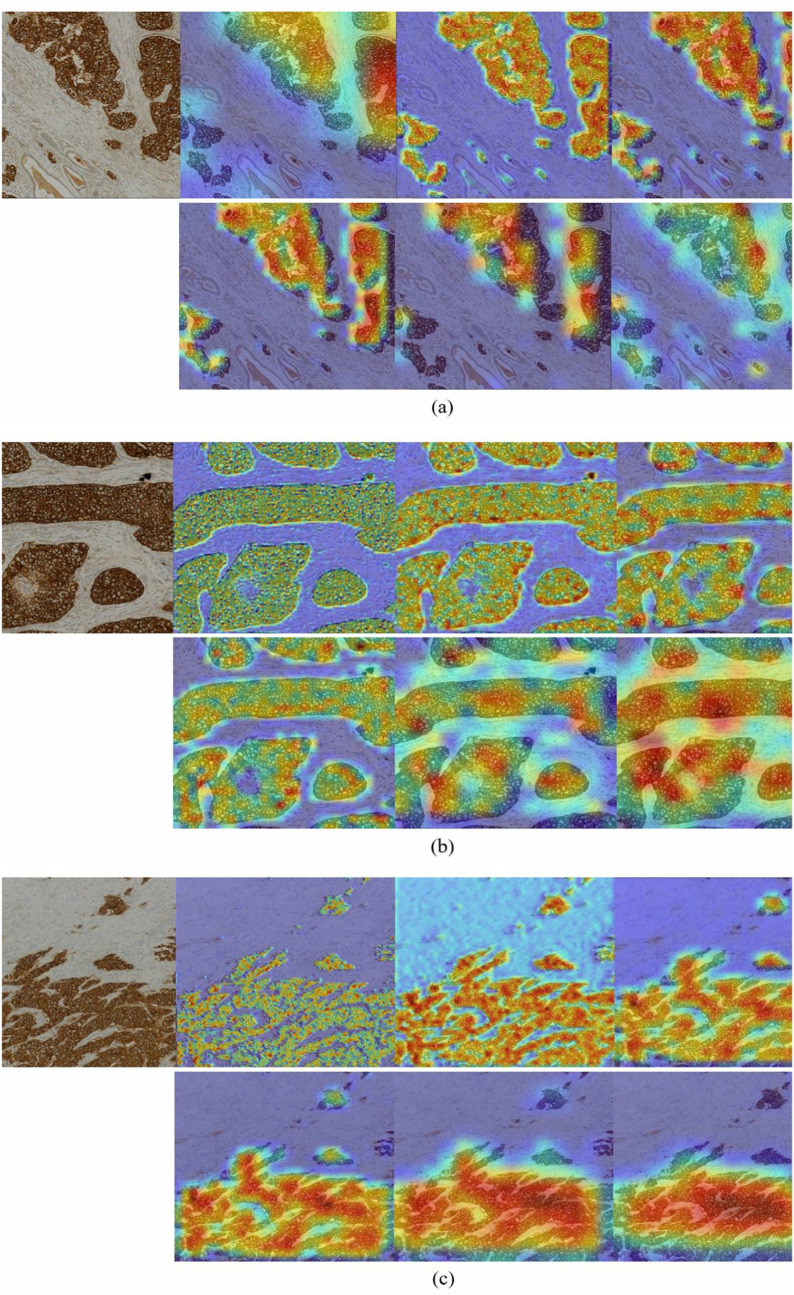
HER2 positive IHC images along with their Grad-CAM heatmaps from the top Conv2D layers of blocks 1 through 6 (starting from top-left) of the EfficientNetV2B0 network pretrained on ImageNet.

**Fig 10:**
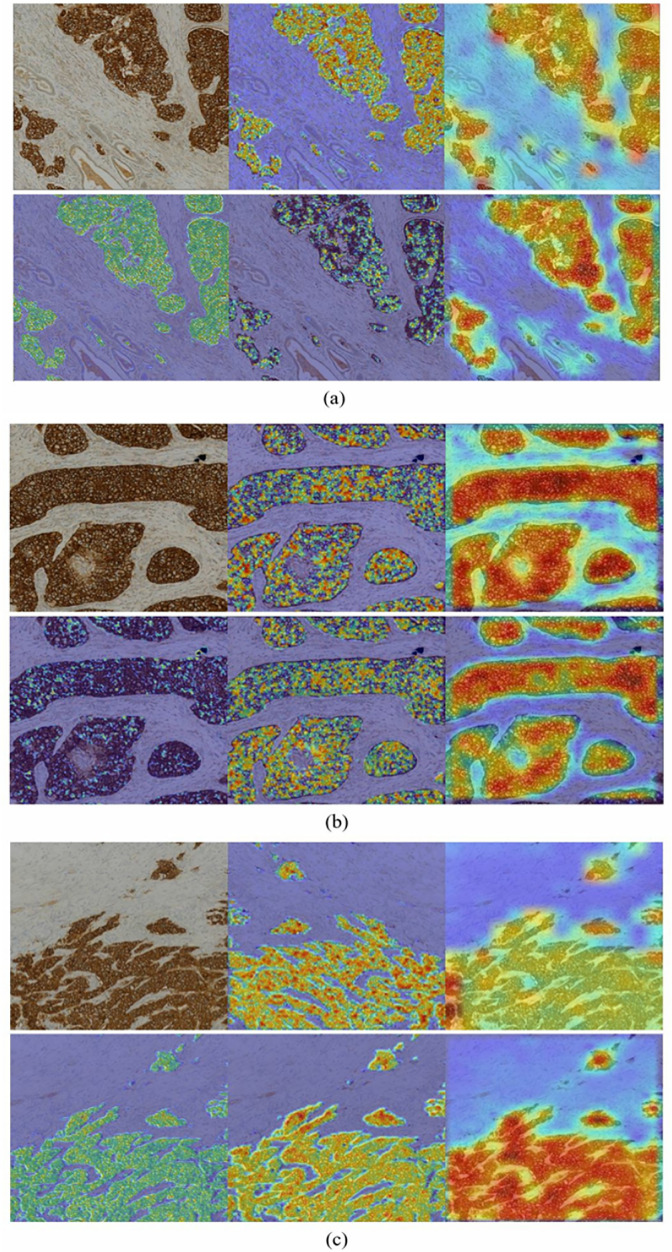
HER2 positive IHC images along with their Grad-CAM heatmaps for the proposed ATHER2 network. The top row includes heatmaps generated from the L1 and L4 Conv2D layers in ATHER2’s left branch, whereas the bottom row includes the heatmaps generated from ATHER2’s R1, R3, and R6 Conv2D layers in the network’s right branch.

**Grad-CAM heatmaps:** Grad-CAM was implemented to visualize the parts within the input images that affected the final decision made by the deep networks. The ASCO/CAP guidelines use staining intensity and completeness of the circumferential stained cell membrane to discriminate HER2 0, 1+ , 2+ , and 3+ cases within IHC stained images [9]. In HER2-positive patches, strong complete membranous staining encompassing the cells (appearing as dark brown circles within the IHC images) is thus the key feature that deep networks should identify for reliable HER2 scoring [16]. Several sample images were selected from the test dataset to evaluate the Grad-CAM performance for the two presented lightweight networks. Figs 9 and 10 illustrate the generated heatmaps for three sample HER2-positive IHC stained images using EfficientNetV2B0 and ATHER2, respectively. For EfficientNetV2B0, Grad-CAM was applied to the final convolutional layer of each of its six blocks, which corresponds to the final convolutional layer of each subnetwork. For ATHER2, Grad-CAM was applied to every convolutional layer in its two different branches, which for the left branch are layers L1 & L4 and for the right branch are layers R1, R3, & R6 as denoted in Table 2.

**Expert pathologist evaluation of the generated heatmaps:** All heatmaps were evaluated and approved by a specialist BC pathologist with over 10 years of experience. The pathologist confirmed that the generated heatmaps efficiently highlighted the HER2-positive structures within the IHC stained image patches (designated as the dark brown regions) for both the presented lightweight networks. For EfficientNetV2B0, the middle layer heatmaps are generally shown to better capture the HER2-positve related structures as compared to its top layers. These findings support the block pruning results which showed that removing the top layers of EfficientNetV2B0 had a minimal effect on its overall performance. As for the ATHER2 network, the convolutional filters incrementally start identifying relevant structures as images are derived from deeper network layers. Overall, both proposed lightweight models reliably capture the HER2 related structures within the IHC

#### HER2 binary classification using H&E images (BCI Dataset).

In this section, the performance of the presented lightweight networks is assessed for binary HER2 classification using H&E stained images. The original EfficientNetV2B0 network and the PrunEff4 subnetwork pretrained on ImageNet are compared to the customized ATHER2 network for HER2 classification. As in the above subsection, two cases were considered for the ATHER2 network which are (1) freshly initialized and (2) domain-specific pretraining on the balanced BreakHis dataset.

Table 10 summarizes the results of the different HER2 classification experiments using H&E images. An interesting observation is that the domain-specific pretraining of ATHER2 resulted in significant performance improvement compared to the freshly initialized case (~6%), whereas it was earlier shown that no improvement was achieved when using the IHC images (Table 9). HER2 related structures are barely prominent in H&E images, unlike in IHC images in which HER2 related features are clearly visible. Experimental results thus indicate that domain-specific pretraining can be more beneficial in more challenging classification tasks in which the features are more subtle requiring advanced techniques for enhancing performance.

**Table 10:** HER2 2-class classification performance using H&E images (BCI dataset).

	Pretraining	Frozen Layers	Accuracy	Precision	Recall	F1-Score	AUC
**EfficientNetV2B0**	**ImageNet**	0	98.61	97.83	98.90	98.36	99.78
**PrunEff4**	**ImageNet**	0	97.69	97.78	96.70	97.24	99.72
**ATHER2**	**None**	--	90.91	85.71	94.74	90.00	96.29
	**BreakHis**	0	96.36	96.77	94.74	95.74	98.83
	**BreakHis**	6	97.27	95.88	97.89	98.36	99.33

As with the results in the previous subsection, EfficientNetV2B0 and PrunEff4 were found to give somewhat close accuracies of 98.61% and 97.69%, respectively. In addition, it is shown that ATHER2 with domain-specific pretraining resulted in an accuracy of 97.27% which is comparable to both EfficientNetV2B0 and PrunEff4 pretrained on ImageNet, yet using a significantly less computationally expensive network. Raghu et al. [35] have previously shown that for retinal and chest X-Ray images, lightweight customized models pretrained on domain-specific datasets offered similar performance to the ResNet50 pretrained on ImageNet. Our findings for HER2 classification from histopathological images are thus in agreement with other research in the medical disease classification field.

In clinical practice, IHC staining of histopathological images is the gold standard for HER2 scoring. However, given that the majority of pathological slides are routinely H&E stained for the initial BC detection, directly using H&E stained images for HER2 scoring would enable rapid and cost-effective diagnosis. Recently, several studies have investigated the usefulness of H&E stained images for deep learning-based HER2 scoring and have reported promising results [4,7,23,49]. Experiments performed in this section also support this finding. The difference between the approach adopted in this work and previous literature is the consideration of standard network pruning and domain-specific pretraining to develop lightweight networks, both which were shown to give reliable performance whether IHC or H&E images were input to the network.

#### HER2 multiclass classification using the BCI Dataset.

In this section, we consider the borderline HER2 (2+) case, which is the most challenging to detect by pathologists, in addition to the three other cases. Primarily, the histopathological images were classified into 3 classes: HER2 negative (0/1+), borderline (2+), and positive (3+). The 3-class HER2 classification was performed as the ASCO/CAP 2018 HER2 testing guidelines suggested grouping together scores 0 and 1+ as HER2 negative. Advancements in BC medical treatments have shown that HER2 score 0 and score 1 + would benefit from different treatment options [50]. In this case, it would be more convenient to categorize them into two separate classes. Consequently, the histopathological images were also classified into four HER2 classes: score 0, score 1+ , score 2+ , and score 3+ .

Tables 11 and 12 summarize the results of the 3-class and 4-class HER2 classifications, respectively. For both experiments, EfficientNetV2B0 and PrunEff4 resulted in accuracies that exceeded 97% for the IHC images and was in the range between 95% and 97% for the H&E images. Interestingly, the performance of the pretrained ATHER2 network was very similar for both types of stained images. ATHER2 resulted in accuracies of 97.54% and 96.01% using IHC images for 3- and 4- class classification, respectively. As for H&E images, ATHER2 pretrained on BreakHis resulted in ~96% accuracy for both multiclass classification experiments.

**Table 11:** HER2 3-class classification performance using IHC and H&E images (BCI dataset).

	Pretraining	# of Frozen Layers	IHC Images			H&E Images		
			Accuracy	F1-Score	AUC	Accuracy	F1-Score	AUC
**EfficientNetV2B0**	ImageNet	0	97.14	97.14	99.81	95.83	95.69	99.37
**PrunEff4**	**ImageNet**		**97.14**	**97.26**	**99.87**	**96.09**	**95.66**	**99.55**
**ATHER2**	None	--	91.65	91.65	98.62	81.57	80.20	94.18
	BreakHis	0	97.30	97.30	99.53	93.61	93.48	98.63
	**BreakHis**	**6**	**97.54**	**97.14**	**99.65**	**95.58**	**95.69**	**99.34**

**Table 12:** HER2 4-class classification performance using IHC and H&E images (BCI dataset).

	Pretraining	# of Frozen Layers	IHC Images			H&E Images		
			Accuracy	F1-Score	AUC	Accuracy	F1-Score	AUC
**EfficientNetV2B0**	ImageNet	0	97.40	97.52	99.90	95.83	95.81	99.49
**PrunEff4**	**ImageNet**		**98.44**	**98.57**	**99.72**	**97.14**	**97.11**	**99.36**
**ATHER2**	None	--	61.43	56.89	86.50	51.60	31.73	77.59
	BreakHis	0	93.12	92.72	98.81	95.09	94.54	99.28
	**BreakHis**	**6**	**96.01**	**95.94**	**99.57**	**96.07**	**96.26**	**99.59**

Interestingly, domain-specific pretraining is shown to significantly increase performance regardless of the considered histopathological image type for multiclass classification. More substantial improvements, however, are observed for the H&E images as compared to the IHC images. Additionally, domain-specific pretraining led to more substantial performance gains when more HER2 classes were considered, i.e., 4-classes (Table 12) vs. 3-classes (Table 11) vs. 2-classes (Table 9). In this work, the role of domain-specific pretraining is thus shown to be more crucial with the increased complexity of the classification task, e.g., using the H&E images and/or increasing the number of classes.

**Fig 11:**
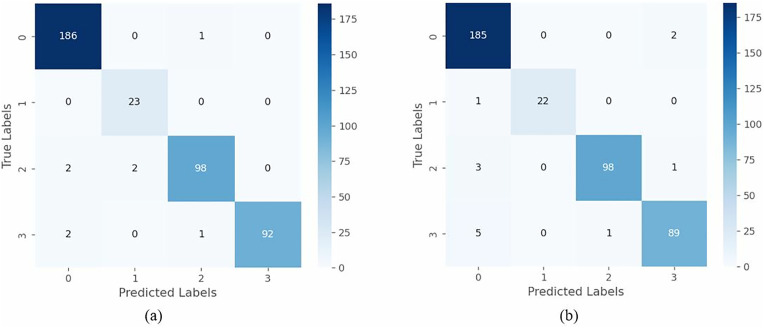
Confusion matrices for the proposed PrunEff4 considering (a) IHC and (b) H&E images.

**Fig 12:**
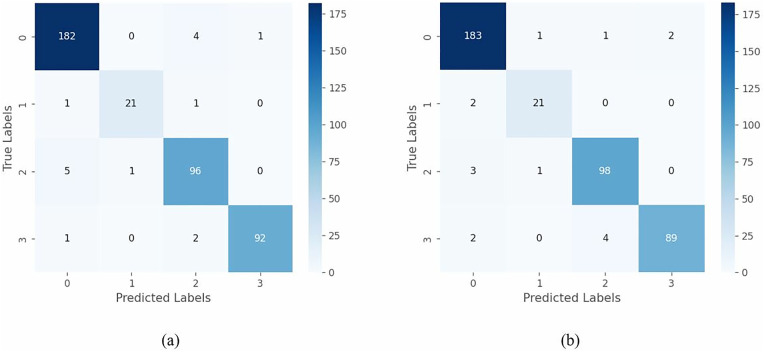
Confusion matrices for the proposed ATHER2 considering (a) IHC and (b) H&E images.

In the previous experiments, the two proposed lightweight models (PrunEff4 and ATHER2) were shown to give reliable performance for both IHC and H&E images. Figs 11 and 12 illustrate the confusion matrices generated for the two models considering the IHC and H&E images, respectively. Additionally, Tables 13 and 14 summarize the per-class classification performance for the two networks, considering the IHC and H&E images, respectively. In most cases, classes 0 and 3+ were the easiest to detect, whereas classes 1+ and 2+ were the more challenging. Interestingly, scores 1+ and 2+ gave better performance when H&E images were considered for training the PrunEff4 network.

**Table 13:** PrunEff4 per class performance metrics (BCI dataset).

Class	IHC Images			H&E Images		
	Precision	Recall	F1-score	Precision	Recall	F1-score
0	0.98	0.99	0.99	0.95	0.99	0.97
1+	0.92	1.00	0.96	1.00	0.96	0.98
2+	0.98	0.96	0.97	0.99	0.96	0.98
3+	1.00	0.97	0.98	0.97	0.94	0.95

**Table 14:** ATHER2 per class performance metrics (BCI dataset).

Class	IHC Images			H&E Images		
	Precision	Recall	F1-score	Precision	Recall	F1-score
0	0.96	0.97	0.97	0.96	0.98	0.97
1+	0.95	0.91	0.93	0.91	0.91	0.91
2+	0.93	0.94	0.94	0.95	0.96	0.96
3+	0.99	0.97	0.98	0.98	0.94	0.96

Finally, the Matthews Correlation Coefficient (MCC) statistical measure was computed to further evaluate the performance of the proposed networks (Table 15). MCC is particularly useful in the case of imbalanced data, where it provides a more informative score than accuracy or F1-score [51]. For both networks, the MCC ranged from 0.93 to 0.97, indicating the proposed reliable performance of the proposed networks despite the slight imbalance present in the BCI dataset.

**Table 15:** Matthews Correlation Coefficient (MCC) for the proposed models.

	IHC Images	H&E Images
**PrunEff4**	0.97	0.95
**ATHER2**	0.93	0.94

Tables 16 compares the performance of PrunEff4 and ATHER2 networks to several deep learning methods from literature considering the BCI dataset. ATHER2 and the PrunEff4 networks both gave accuracies that ranged from 95% to 98% for the multiclass HER2 classification using either H&E or IHC images. The presented networks thus outperform other methods from literature by 2% to 15%. Table 17 summarizes the results of several standard networks that were reimplemented by the authors to compare their performance to PrunEff4 and ATHER2 under similar experimental conditions. Results show that the presented lightweight models performed just as well or better than all the reimplemented models, regardless of the considered histopathological image type. Experimental results thus indicate the suitability of the proposed lightweight networks for HER2 classification from both IHC and H&E stained images. In addition, results demonstrate that well-designed lightweight models can achieve reliable performance that is comparable to state-of-the-art networks with the added advantage of being suitable for deployment on resource-constrained devices.

**Table 16:** Performance comparison with other methods from literature (BCI dataset – 4 classes).

Reference	Year	Architecture	HE/IHC Images	Accuracy
**Mridha et al.** [19]	2022	convoHER2 (based on InceptionV3)	IHC	88.0
			H&E	85.0
**Shovon et al.** [4]	2022	Xception	H&E	70.0
		InceptionResNetV2		71.0
		HE-HER2Net (based on Xception)		87.0
**Wang et al.** [7]	2023	ResNet101	H&E	72.77
		DenseNet161		80.96
		InceptionV3		84.23
		HAHNet (based on InceptionV3 and attention modules)		93.65
**Proposed**	2024	PrunEff4	IHC	98.44
			H&E	97.14
		ATHER2	IHC	96.01
			H&E	96.07

**Table 17:** Performance comparison with several reimplemented standard networks (BCI dataset – 4 classes).

Architecture	Parameters (million)	FLOPs (x10^9^)	IHC		H&E	
			Accuracy	AUC	Accuracy	AUC
**ATHER2**	0.35	0.389	96.01	99.72	96.07	99.59
**PrunEff4**	1.5	0.885	98.44	99.80	97.17	99.36
**MobileNetV2**	3.5	0.615	95.57	99.75	94.53	99.42
**NASNetMobile**	5.3	1.15	92.45	98.60	86.46	97.34
**EfficientNetV2B0**	7.2	1.46	97.40	99.90	95.83	99.49
**InceptionV3**	23.9	5.70	95.57	99.30	91.67	97.85
**ResNet50V2**	25.7	6.99	96.88	99.66	92.45	97.83

#### HER2 multiclass classifications using the Warwick dataset.

The Warwick dataset, which has not been previously seen by the proposed networks, is considered to test the generalization capability of the models. Two experiments were performed depending on how the samples were divided among the train and test datasets. In Warwick Exp#1, the train and test datasets were randomly selected thus included samples from the same subjects. In Warwick Exp#2, the train and test datasets were subject-oriented meaning that they had samples from completely different subjects (as was the case with the BCI dataset used in all previous experiments). Warwick Exp#2 is considered a more generalized and realistic experiment since, samples from previous patients are typically employed to diagnose new patients.

For the ATHER2 network, the freshly initialized and domain-specific pretraining results are presented in order to demonstrate the effect of the domain-specific pretraining on the network’s performance. In addition, the 2-class BreakHis dataset (BC H&E images) and the 2-class BCI dataset (HER2 IHC images) were compared for the domain-specific pretraining of the ATHER2 network. The aim of this analysis is to investigate the benefit of using a closely related dataset (H&E images) as compared to using a dataset from the exact domain (IHC images) for pretraining ATHER2 when targeting the Warwick dataset (IHC images).

Tables 18 and 19 summarize the 3-class (0/1+ vs. 2+ vs 3+) and 4-class (0 vs. 1+ vs. 2+ vs 3+) HER2 classification experiments, respectively. Several observations can be drawn from these experiments:

**Table 18:** HER2 3-class classification performance using IHC images (Warwick dataset).

	Pretraining	Frozen Layers	Warwick Exp#1			Warwick Exp#2		
			Accuracy	F1-Score	AUC	Accuracy	F1-Score	AUC
**EfficientNetV2B0**	ImageNet	0	**98.26**	**98.26**	**99.92**	**97.57**	**96.10**	**99.58**
**PrunEff4**	ImageNet		98.26	98.26	99.88	96.18	96.00	99.34
**ATHER2**	None	--	90.67	90.67	98.00	92.64	92.77	92.64
	BreakHis	0	97.00	97.00	99.74	**97.32**	**97.32**	**99.56**
	BreakHis	6	97.00	97.00	99.77	96.32	96.32	99.82
	BCI	0	**98.00**	**98.00**	**99.66**	95.32	95.32	99.00
	BCI	6	97.67	97.67	99.83	96.66	96.66	99.64

**Table 19:** HER2 4-class classification performance using IHC images (Warwick dataset).

	Pretraining	Frozen Layers	Warwick Exp#1			Warwick Exp#2		
			Accuracy	F1-Score	AUC	Accuracy	F1-Score	AUC
**EfficientNetV2B0**	ImageNet	0	**97.57**	**97.57**	**99.91**	**96.53**	**96.53**	**99.54**
**PrunEff4**	ImageNet		96.88	96.88	99.92	96.18	96.18	99.37
**ATHER2**	None	--	85.33	83.81	97.22	91.97	91.31	98.64
	BreakHis	0	97.33	97.33	99.81	**97.66**	**97.66**	**99.65**
	BreakHis	6	97.33	97.33	99.73	96.66	96.66	99.32
	BCI	0	97.67	97.67	99.81	96.66	96.66	99.32
	BCI	6	**97.67**	**97.67**	**99.82**	96.66	96.66	99.37

a) Warwick Exp#1 performance exceeded that of the Warwick Exp#2 by ~1-3%.
  - These results are expected since the former considers the case where the train and test dataset have samples from the same subjects, thus leading to somewhat overoptimized results.
b) For both experiments, the performance of the pretrained EfficientNetV2B0 and PrunEff4 networks were quite close despite the enormous difference in trainable parameters (7.2 million vs. 1.5 million).
  - These results demonstrate the advantages of network pruning in achieving reliable performance with the added benefit of reduced network size.
c) For both experiments, domain-specific pretraining of ATHER2 boosted performance to be quite similar to that of both EfficientNetV2B0 and PrunEff4.
  - These results indicate that pretraining with a domain related dataset substantially enhances performance of lightweight customized deep networks.
d) For both experiments, pretraining ATHER2 with the BCI dataset (IHC images) gave better results than pretraining with the BreakHis dataset (H&E images). Furthermore, performance improvement was more prominent within more challenging experiments, i.e., the increase in performance was more relevant for the 4-class classification than for the 3-class classification.
  - These results indicate that domain-specific training gave better results with increased similarity between the pretraining dataset and target datasets, and that it was more useful with the increase in the number of classes.

Overall, the Warwick experiments showed that the two presented lightweight networks generalized well to unseen data, indicating their reliability for automatic HER2 classification from histopathological images. Table 20 summarizes the performance of several deep learning methods from literature for the Warwick IHC dataset. Results indicate that the proposed lightweight networks outperform previous approaches by up to 6%. Table 21 compares the performance of the two proposed lightweight models to several reimplemented lightweight and state-of-the-art standard networks pretrained on ImageNet. The reimplementation of the networks provides a robust comparison, since experiments were performed under identical conditions. The two lightweight models introduced in this study are shown to achieve significantly better performance as compared to the lightweight MobileNetV2 and NASNetMobile. Additionally, PrunEff4 and ATHER2 gave comparative performance to the state-of-the-art InceptionV3 and ResnNet50V2, with the added advantage of significantly reduced number of parameters.

**Table 20:** Performance comparison with other methods from literature (Warwick dataset – IHC images).

Reference	Year	Architecture	# of WSIs	# of Classes	Accuracy
**Tewary et al.** [14]	2021	NASNetMobile	40	3	75.00
		MobileNetV2			81.00
		ResNet50			87.00
		VGG16			92.00
		VGG19			93.00
**Tewary et al.** [17]	2022	Xception	40	3	95.00
		DL Model (AutoIHCNet)			96.00
**Kabir et al.** [16]	2024	GoogleNet	77	4	85.73
		MobileNetV2			88.73
		DenseNet201			90.66
		Vision Transformer (ViT)			91.15
**Proposed**	2024	PrunEff4	52	4	96.18
		ATHER2			97.66

**Table 21:** Performance comparison with several reimplemented standard networks (Warwick dataset – IHC images - 4 classes).

Architecture	Parameters (x10^6^)	FLOPs (x10^9^)	Accuracy	AUC
**ATHER2**	0.35	0.389	97.66	99.65
**PrunEff4**	1.5	0.885	96.18	99.37
**MobileNetV2**	3.5	0.615	93.40	98.92
**NASNetMobile**	5.3	1.15	82.29	95.77
**EfficientNetV2B0**	7.2	1.46	96.53	99.54
**InceptionV3**	23.9	5.70	96.53	99.65
**ResNet50V2**	25.7	6.99	94.79	99.21

## Conclusions

H&E images have the advantage of being more readily available than IHC images due to being routine procedure in BC diagnosis. In addition, they are less expensive and require less sophisticated equipment. These benefits have motivated researchers to exploit the strengths of deep learning to automatically learn relevant structures within the H&E images for reliable HER2 scoring. However, since HER2 related structures are not as prominent in H&E images as they are in IHC images, complex deep networks have been predominantly used in literature for this task. Nevertheless, complex deep networks are not well-suited for deployment on resource-constrained devices commonly found in low-resource clinical settings — such as in rural hospitals, mobile labs, or low-income regions. In the present study, two lightweight models were presented for automatic HER2 scoring using IHC or H&E images: ATHER2 and PrunEff4.

ATHER2 is a small, customized network (~345k parameters) that was built using different sized convolutional filters along with CBAM attention to better capture relevant features in the histopathological images. Domain-specific pretraining of the customized ATHER2 network was shown to significantly improve its performance, specifically for more complex classification tasks such as when H&E images were used for HER2 scoring or for increased number of classes. In addition, performance enhancement was more prominent when both the pretraining and target datasets considered the same type of images. An interesting finding of this work is thus the merit of domain adaptation to facilitate customized network training, improve overall performance, and enhance its generalization capabilities.

PrunEff4 is a pruned version of the standard lightweight EfficientNetV2B0 in which the two top blocks were removed to reduce its computational complexity while still achieving reliable performance. PrunEff4 was devised based on a comprehensive analysis in which top blocks from the EfficientNetV2B0 were subsequently removed and the effect on performance was closely monitored. Block pruning led to a reduction in the number of parameters from ~7.2 million to ~1.5 million while maintaining reliable HER2 scoring performance. Off-the-shelf standard networks, e.g., EfficientNets, were mainly developed and tested considering complex tasks such as the classification of objects into 1,000 different classes. In many real-world applications, the full complexity of the standard networks is thus unnecessary due to the comparatively simpler nature of the tasks. Block pruning was proposed in this work to retain the strengths of the standard EfficientNetV2B0 network while further reducing its computational cost.

Most previous works considered either IHC or H&E images for HER2 scoring, with only a few studies comparing the performance of both types of histopathological images for their proposed methods. In this work, the two proposed lightweight models were compared for both IHC and H&E images demonstrating reliable performance in either case. Two different histopathological datasets were utilized for the development and testing of the proposed networks to better evaluate their generalization capabilities. In all experiments, both models achieved accuracies ranging from 97% to 100% for binary classifications and from 95.5% to 98.5% for multiclass classifications. The two proposed lightweight models achieved state-of-the-art performance, outperforming several standard and customized deep networks from the literature considering IHC images, as well as H&E images in which HER2-related structures are not readily prominent.

In conclusion, this study demonstrates the efficacy of domain-pretraining of customized networks and block pruning of standard networks for developing lightweight models that are suitable for resource-constrained devices. Additionally, H&E stained images were shown to be reliable for deep learning-based HER2 scoring, achieving comparable results to when IHC stained images were utilized. Since H&E staining is considered routine procedure for all suspicious cancer biopsy samples, using them for automatic HER2 classification can offer significant cost and time savings for both the patients and the medical practitioners.

Our preliminary findings using the two lightweight networks PrunEff4 and ATHER2 are promising. Nevertheless, there are a few limitations that can be addressed in future work. Collaborations with multiple medical centers can be established to evaluate the performance of the presented models across different image sources. Multimodal data combining imaging features with pathology reports can be considered to further enhance the model’s robustness and accuracy. Additionally, the presented lightweight models can be deployed on edge devices to evaluate their real-time performance.
